# (2*E*,4*E*)-*N*-Benzyl-2-cyano-5-phenyl­penta-2,4-dienamide

**DOI:** 10.1107/S1600536811036397

**Published:** 2011-09-14

**Authors:** Xin-Li Li

**Affiliations:** a College of Chemistry and Chemical Engineering, China West Normal University, Nanchong 637002, People’s Republic of China

## Abstract

In the title compound, C_19_H_16_N_2_O, the mol­ecule adopts an *E* configuration about the two C=C double bonds. The dihedral angle between the phenyl rings is 88.89 (8)°. In the crystal, mol­ecules are linked by inter­molecular N—H⋯N and C—H⋯O hydrogen bonds into chains running parallel to [130].

## Related literature

For the use of malononitrile-containing compounds as building blocks in organic synthesis, see: Liu *et al.* (2002 ▶); Sepiol & Milart (1985 ▶); Zhang *et al.* (2003 ▶). For a related structure, see: Kang & Chen (2009 ▶).
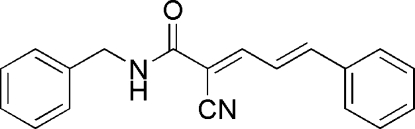


## Experimental

### 

#### Crystal data

C_19_H_16_N_2_O
*M* *_r_* = 288.34
Monoclinic, 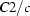

*a* = 19.5823 (19) Å
*b* = 5.6386 (8) Å
*c* = 28.450 (3) Å
β = 94.912 (9)°
*V* = 3129.8 (6) Å^3^
*Z* = 8
Cu *K*α radiation
μ = 0.61 mm^−1^
*T* = 291 K
0.30 × 0.24 × 0.20 mm

#### Data collection

Oxford Diffraction Xcalibur Sapphire3 Gemini ultra diffractometer
Absorption correction: multi-scan (*CrysAlis PRO*; Oxford Diffraction, 2009 ▶) *T*_min_ = 0.839, *T*_max_ = 0.889
6165 measured reflections
2789 independent reflections
1843 reflections with *I* > 2σ(*I*)
*R*_int_ = 0.027

#### Refinement

*R*[*F* ^2^ > 2σ(*F* ^2^)] = 0.047
*wR*(*F* ^2^) = 0.137
*S* = 1.05
2789 reflections
199 parameters
H-atom parameters constrained
Δρ_max_ = 0.12 e Å^−3^
Δρ_min_ = −0.13 e Å^−3^

### 

Data collection: *CrysAlis PRO* (Oxford Diffraction, 2009 ▶); cell refinement: *CrysAlis PRO*; data reduction: *CrysAlis PRO*; program(s) used to solve structure: *SHELXS97* (Sheldrick, 2008 ▶); program(s) used to refine structure: *SHELXL97* (Sheldrick, 2008 ▶); molecular graphics: *ORTEP-3* (Farrugia, 1997 ▶); software used to prepare material for publication: *SHELXL97*.

## Supplementary Material

Crystal structure: contains datablock(s) global, I. DOI: 10.1107/S1600536811036397/rz2635sup1.cif

Structure factors: contains datablock(s) I. DOI: 10.1107/S1600536811036397/rz2635Isup2.hkl

Supplementary material file. DOI: 10.1107/S1600536811036397/rz2635Isup3.cml

Additional supplementary materials:  crystallographic information; 3D view; checkCIF report

## Figures and Tables

**Table 1:** Hydrogen-bond geometry (Å, °)

*D*—H⋯*A*	*D*—H	H⋯*A*	*D*⋯*A*	*D*—H⋯*A*
N2—H2⋯N1^i^	0.86	2.25	3.037 (2)	152
C7—H7⋯O1^ii^	0.93	2.39	3.280 (2)	160

Symmetry codes: (i) 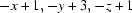; (ii) 
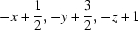
.

**Fig. 1.**
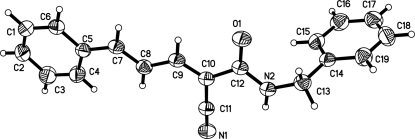
The molecular structure of (I) with 30% probability displacement ellipsoids.

## supplementary crystallographic information

### Comment

The chemistry of ylidene malononitrile have been studied extensively. From ring closure reactions, compounds containing newly formed five or six-membered rings, such as indans (Zhang *et al.*, 2003), naphthalenes (Liu *et al.*, 2002) and benzenes (Sepiol & Milart, 1985) were obtained. Some crystal structures involving ylidene malononitrile groups have been published, including a recent report from our laboratory (Kang & Chen, 2009). As a part of our interest in the synthesis of complex ring systems, we investigated the title compound, (I), which is a diene reagent in the Diels-Alder reaction. We report herein the crystal structure of the title compound.

The molecular structure of (I) is shown in Fig. 1. Bond lengths and angles are normal. The two phenyl rings are almost perpendicular, forming a dihedral angle of  88.89 (8)°. Both C═C double bonds display an *E* configuration. In the crystal packing, molecules are connected by intermolecular N—H···N and C—H···O hydrogen bonds (Table 1) to form chains running parallel to the [130] direction.

### Experimental

(2*E*,4*E*)-Ethyl 2-cyano-5-phenylpenta-2,4-dienoate (0.454 g, 2 mmol) and phenylmethanamine (0.214 g, 2 mmol) were dissolved in 2-propanol (2 ml). To the solution was added piperidine (0.017 g, 0.2 mmol), then the mixture was stirred for 24 h at 298 K and filtered to obtain a white solid. Recrystallization from hot ethanol afforded the pure compound. Single crystals of (I) suitable for X-ray analysis were obtained by slow evaporation of the ethanol solvent.

### Refinement

All H atoms were placed in calculated positions, with C—H = 0.93–0.97 Å, N—H = 0.86 Å, and refined using a riding model, with *U*_iso_(H) = 1.2*U*_eq_(C, N).

### Crystal data

**Table:**

C_19_H_16_N_2_O	*F*(000) = 1216
*M**_r_* = 288.34	*D*_x_ = 1.224 Mg m^−^^3^
Monoclinic, *C*2/*c*	Cu *K*α radiation, λ = 1.54184 Å
Hall symbol: -C 2yc	Cell parameters from 1369 reflections
*a* = 19.5823 (19) Å	θ = 3.1–69.3°
*b* = 5.6386 (8) Å	µ = 0.61 mm^−^^1^
*c* = 28.450 (3) Å	*T* = 291 K
β = 94.912 (9)°	Needle, yellow
*V* = 3129.8 (6) Å^3^	0.30 × 0.24 × 0.20 mm
*Z* = 8	

### Data collection

**Table:**

Oxford Diffraction Xcalibur Sapphire3 Gemini ultra diffractometer	2789 independent reflections
Radiation source: Enhance Ultra (Cu) X-ray Source	1843 reflections with *I* > 2σ(*I*)
mirror	*R*_int_ = 0.027
Detector resolution: 15.9149 pixels mm^-1^	θ_max_ = 67.1°, θ_min_ = 3.1°
ω scans	*h* = −23→21
Absorption correction: multi-scan (*CrysAlis PRO*; Oxford Diffraction, 2009)	*k* = −4→6
*T*_min_ = 0.839, *T*_max_ = 0.889	*l* = −33→31
6165 measured reflections	

### Refinement

**Table:**

Refinement on *F*^2^	Primary atom site location: structure-invariant direct methods
Least-squares matrix: full	Secondary atom site location: difference Fourier map
*R*[*F*^2^ > 2σ(*F*^2^)] = 0.047	Hydrogen site location: inferred from neighbouring sites
*wR*(*F*^2^) = 0.137	H-atom parameters constrained
*S* = 1.05	*w* = 1/[σ^2^(*F*_o_^2^) + (0.0532*P*)^2^ + 0.3114*P*] where *P* = (*F*_o_^2^ + 2*F*_c_^2^)/3
2789 reflections	(Δ/σ)_max_ < 0.001
199 parameters	Δρ_max_ = 0.12 e Å^−^^3^
0 restraints	Δρ_min_ = −0.13 e Å^−^^3^

### Special details

**Table:**

Geometry. All e.s.d.'s (except the e.s.d. in the dihedral angle between two l.s. planes) are estimated using the full covariance matrix. The cell e.s.d.'s are taken into account individually in the estimation of e.s.d.'s in distances, angles and torsion angles; correlations between e.s.d.'s in cell parameters are only used when they are defined by crystal symmetry. An approximate (isotropic) treatment of cell e.s.d.'s is used for estimating e.s.d.'s involving l.s. planes.
Refinement. Refinement of *F*^2^ against ALL reflections. The weighted *R*-factor *wR* and goodness of fit *S* are based on *F*^2^, conventional *R*-factors *R* are based on *F*, with *F* set to zero for negative *F*^2^. The threshold expression of *F*^2^ > σ(*F*^2^) is used only for calculating *R*-factors(gt) *etc*. and is not relevant to the choice of reflections for refinement. *R*-factors based on *F*^2^ are statistically about twice as large as those based on *F*, and *R*- factors based on ALL data will be even larger.

### Fractional atomic coordinates and isotropic or equivalent isotropic displacement parameters (Å^2^)

**Table:**

	*x*	*y*	*z*	*U*_iso_*/*U*_eq_	
O1	0.28610 (7)	1.1494 (3)	0.45363 (5)	0.0770 (5)	
N2	0.36754 (9)	1.4308 (3)	0.45249 (6)	0.0650 (5)	
H2	0.4081	1.4745	0.4629	0.078*	
N1	0.50308 (9)	1.2707 (4)	0.53540 (7)	0.0751 (6)	
C10	0.38243 (9)	1.1118 (4)	0.50910 (7)	0.0559 (5)	
C5	0.38487 (10)	0.4455 (4)	0.62469 (7)	0.0596 (5)	
C14	0.33781 (10)	1.5116 (4)	0.36648 (7)	0.0592 (5)	
C11	0.44961 (10)	1.1992 (4)	0.52414 (7)	0.0594 (5)	
C8	0.38929 (10)	0.7868 (4)	0.56840 (7)	0.0627 (6)	
H8	0.4334	0.8274	0.5805	0.075*	
C9	0.35694 (10)	0.9231 (4)	0.53033 (7)	0.0592 (5)	
H9	0.3131	0.8752	0.5190	0.071*	
C12	0.34132 (9)	1.2332 (4)	0.46947 (7)	0.0578 (5)	
C7	0.35797 (10)	0.6024 (4)	0.58708 (7)	0.0609 (5)	
H7	0.3135	0.5711	0.5744	0.073*	
C19	0.31165 (11)	1.6639 (5)	0.33143 (9)	0.0766 (7)	
H19	0.2898	1.8023	0.3397	0.092*	
C13	0.32942 (12)	1.5726 (4)	0.41717 (8)	0.0706 (6)	
H13B	0.3426	1.7370	0.4222	0.085*	
H13A	0.2812	1.5603	0.4221	0.085*	
C4	0.45148 (11)	0.4587 (5)	0.64515 (9)	0.0799 (7)	
H4	0.4805	0.5765	0.6355	0.096*	
C15	0.36965 (11)	1.3083 (4)	0.35289 (8)	0.0718 (6)	
H15	0.3875	1.2015	0.3756	0.086*	
C6	0.34295 (11)	0.2684 (4)	0.64009 (8)	0.0679 (6)	
H6	0.2978	0.2571	0.6271	0.081*	
C1	0.36710 (12)	0.1092 (5)	0.67424 (8)	0.0792 (7)	
H1	0.3383	−0.0082	0.6842	0.095*	
C2	0.43355 (13)	0.1235 (5)	0.69361 (9)	0.0847 (7)	
H2A	0.4501	0.0138	0.7162	0.102*	
C18	0.31711 (13)	1.6154 (6)	0.28437 (9)	0.0899 (8)	
H18	0.2986	1.7197	0.2614	0.108*	
C16	0.37529 (14)	1.2612 (5)	0.30529 (10)	0.0889 (8)	
H16	0.3968	1.1232	0.2965	0.107*	
C3	0.47553 (12)	0.3004 (6)	0.67950 (10)	0.0923 (9)	
H3	0.5202	0.3133	0.6932	0.111*	
C17	0.34949 (15)	1.4158 (6)	0.27158 (10)	0.0915 (8)	
H17	0.3540	1.3848	0.2399	0.110*	

### Atomic displacement parameters (Å^2^)

**Table:**

	*U*^11^	*U*^22^	*U*^33^	*U*^12^	*U*^13^	*U*^23^
O1	0.0564 (8)	0.0861 (12)	0.0854 (11)	−0.0154 (8)	−0.0120 (7)	−0.0014 (9)
N2	0.0644 (10)	0.0644 (12)	0.0641 (11)	−0.0098 (9)	−0.0066 (8)	−0.0037 (9)
N1	0.0593 (11)	0.0816 (14)	0.0830 (13)	−0.0103 (10)	−0.0031 (9)	−0.0061 (11)
C10	0.0512 (10)	0.0589 (13)	0.0574 (11)	−0.0039 (9)	0.0046 (9)	−0.0090 (10)
C5	0.0566 (11)	0.0641 (13)	0.0577 (12)	−0.0048 (10)	0.0033 (9)	−0.0068 (11)
C14	0.0547 (11)	0.0582 (13)	0.0638 (13)	−0.0093 (10)	0.0003 (9)	−0.0040 (11)
C11	0.0554 (11)	0.0611 (13)	0.0614 (12)	−0.0032 (10)	0.0023 (9)	−0.0057 (11)
C8	0.0519 (11)	0.0733 (15)	0.0623 (13)	−0.0059 (10)	0.0023 (9)	−0.0048 (11)
C9	0.0495 (10)	0.0668 (14)	0.0608 (12)	−0.0053 (10)	0.0029 (9)	−0.0101 (11)
C12	0.0519 (11)	0.0636 (13)	0.0573 (12)	−0.0025 (10)	0.0019 (9)	−0.0085 (11)
C7	0.0511 (11)	0.0680 (14)	0.0629 (12)	−0.0034 (10)	0.0001 (9)	−0.0076 (11)
C19	0.0719 (14)	0.0764 (16)	0.0806 (16)	0.0030 (12)	0.0017 (12)	0.0066 (14)
C13	0.0757 (14)	0.0616 (14)	0.0729 (15)	0.0023 (11)	−0.0032 (11)	−0.0045 (12)
C4	0.0620 (13)	0.0937 (19)	0.0823 (16)	−0.0152 (13)	−0.0035 (12)	0.0148 (15)
C15	0.0827 (15)	0.0593 (14)	0.0733 (15)	−0.0022 (12)	0.0069 (12)	−0.0006 (12)
C6	0.0634 (12)	0.0743 (15)	0.0650 (14)	−0.0088 (11)	−0.0009 (10)	−0.0010 (12)
C1	0.0856 (16)	0.0811 (17)	0.0708 (15)	−0.0211 (13)	0.0057 (12)	0.0018 (14)
C2	0.0906 (17)	0.0924 (19)	0.0690 (15)	−0.0053 (15)	−0.0060 (13)	0.0134 (15)
C18	0.0853 (17)	0.109 (2)	0.0735 (17)	−0.0067 (16)	−0.0059 (13)	0.0159 (17)
C16	0.110 (2)	0.0765 (18)	0.0836 (19)	−0.0086 (15)	0.0259 (15)	−0.0155 (16)
C3	0.0691 (14)	0.114 (2)	0.0909 (19)	−0.0113 (15)	−0.0134 (13)	0.0168 (18)
C17	0.105 (2)	0.101 (2)	0.0694 (16)	−0.0202 (18)	0.0087 (15)	−0.0075 (17)

### Geometric parameters (Å, °)

**Table:**

O1—C12	1.230 (2)	C19—H19	0.9300
N2—C12	1.334 (3)	C13—H13B	0.9700
N2—C13	1.442 (3)	C13—H13A	0.9700
N2—H2	0.8600	C4—C3	1.377 (3)
N1—C11	1.142 (2)	C4—H4	0.9300
C10—C9	1.341 (3)	C15—C16	1.394 (3)
C10—C11	1.435 (3)	C15—H15	0.9300
C10—C12	1.494 (3)	C6—C1	1.377 (3)
C5—C4	1.384 (3)	C6—H6	0.9300
C5—C6	1.387 (3)	C1—C2	1.371 (3)
C5—C7	1.452 (3)	C1—H1	0.9300
C14—C15	1.376 (3)	C2—C3	1.374 (4)
C14—C19	1.381 (3)	C2—H2A	0.9300
C14—C13	1.505 (3)	C18—C17	1.356 (4)
C8—C7	1.340 (3)	C18—H18	0.9300
C8—C9	1.431 (3)	C16—C17	1.361 (4)
C8—H8	0.9300	C16—H16	0.9300
C9—H9	0.9300	C3—H3	0.9300
C7—H7	0.9300	C17—H17	0.9300
C19—C18	1.380 (3)		
			
C12—N2—C13	121.55 (18)	N2—C13—H13A	108.1
C12—N2—H2	119.2	C14—C13—H13A	108.1
C13—N2—H2	119.2	H13B—C13—H13A	107.3
C9—C10—C11	120.34 (19)	C3—C4—C5	121.1 (2)
C9—C10—C12	120.41 (18)	C3—C4—H4	119.4
C11—C10—C12	119.25 (19)	C5—C4—H4	119.4
C4—C5—C6	117.9 (2)	C14—C15—C16	120.5 (2)
C4—C5—C7	123.1 (2)	C14—C15—H15	119.8
C6—C5—C7	118.96 (19)	C16—C15—H15	119.8
C15—C14—C19	117.7 (2)	C1—C6—C5	121.0 (2)
C15—C14—C13	123.3 (2)	C1—C6—H6	119.5
C19—C14—C13	119.0 (2)	C5—C6—H6	119.5
N1—C11—C10	178.8 (2)	C2—C1—C6	120.1 (2)
C7—C8—C9	121.93 (19)	C2—C1—H1	119.9
C7—C8—H8	119.0	C6—C1—H1	119.9
C9—C8—H8	119.0	C1—C2—C3	119.7 (2)
C10—C9—C8	127.54 (19)	C1—C2—H2A	120.1
C10—C9—H9	116.2	C3—C2—H2A	120.1
C8—C9—H9	116.2	C17—C18—C19	120.1 (3)
O1—C12—N2	122.8 (2)	C17—C18—H18	120.0
O1—C12—C10	120.0 (2)	C19—C18—H18	120.0
N2—C12—C10	117.16 (17)	C17—C16—C15	120.5 (3)
C8—C7—C5	127.99 (19)	C17—C16—H16	119.8
C8—C7—H7	116.0	C15—C16—H16	119.8
C5—C7—H7	116.0	C2—C3—C4	120.1 (2)
C18—C19—C14	121.5 (2)	C2—C3—H3	120.0
C18—C19—H19	119.2	C4—C3—H3	120.0
C14—C19—H19	119.2	C18—C17—C16	119.8 (3)
N2—C13—C14	116.66 (19)	C18—C17—H17	120.1
N2—C13—H13B	108.1	C16—C17—H17	120.1
C14—C13—H13B	108.1		
			
C11—C10—C9—C8	0.0 (3)	C19—C14—C13—N2	−169.62 (19)
C12—C10—C9—C8	179.81 (19)	C6—C5—C4—C3	0.4 (4)
C7—C8—C9—C10	179.2 (2)	C7—C5—C4—C3	−177.5 (3)
C13—N2—C12—O1	6.1 (3)	C19—C14—C15—C16	0.5 (3)
C13—N2—C12—C10	−174.48 (19)	C13—C14—C15—C16	179.5 (2)
C9—C10—C12—O1	−5.8 (3)	C4—C5—C6—C1	−0.8 (3)
C11—C10—C12—O1	174.09 (19)	C7—C5—C6—C1	177.2 (2)
C9—C10—C12—N2	174.82 (19)	C5—C6—C1—C2	−0.1 (4)
C11—C10—C12—N2	−5.3 (3)	C6—C1—C2—C3	1.4 (4)
C9—C8—C7—C5	178.3 (2)	C14—C19—C18—C17	−0.8 (4)
C4—C5—C7—C8	−4.3 (4)	C14—C15—C16—C17	0.1 (4)
C6—C5—C7—C8	177.9 (2)	C1—C2—C3—C4	−1.8 (4)
C15—C14—C19—C18	−0.1 (3)	C5—C4—C3—C2	0.9 (4)
C13—C14—C19—C18	−179.2 (2)	C19—C18—C17—C16	1.4 (4)
C12—N2—C13—C14	−89.9 (3)	C15—C16—C17—C18	−1.0 (4)
C15—C14—C13—N2	11.3 (3)		

### Hydrogen-bond geometry (Å, °)

**Table:**

*D*—H···*A*	*D*—H	H···*A*	*D*···*A*	*D*—H···*A*
N2—H2···N1^i^	0.86	2.25	3.037 (2)	152.
C7—H7···O1^ii^	0.93	2.39	3.280 (2)	160

Symmetry codes: (i) −*x*+1, −*y*+3, −*z*+1; (ii) −*x*+1/2, −*y*+3/2, −*z*+1.
